# Terahertz Gas-Phase Spectroscopy Using a Sub-Wavelength Thick Ultrahigh-Q Microresonator

**DOI:** 10.3390/s20103005

**Published:** 2020-05-25

**Authors:** Dominik Walter Vogt, Angus Harvey Jones, Rainer Leonhardt

**Affiliations:** 1 Department of Physics, The University of Auckland, Auckland 1010, New Zealand; ajon083@aucklanduni.ac.nz (A.H.J.); r.leonhardt@auckland.ac.nz (R.L.); 2 The Dodd-Walls Centre for Photonic and Quantum Technologies, Dunedin 9016, New Zealand

**Keywords:** terahertz spectroscopy, microresonator, water vapour sensing

## Abstract

The terahertz spectrum provides tremendous opportunities for broadband gas-phase spectroscopy, as numerous molecules exhibit strong fundamental resonances in the THz frequency range. However, cutting-edge THz gas-phase spectrometer require cumbersome multi-pass gas cells to reach sufficient sensitivity for trace level gas detection. Here, we report on the first demonstration of a THz gas-phase spectrometer using a sub-wavelength thick ultrahigh-Q THz disc microresonator. Leveraging the microresonator’s ultrahigh quality factor in excess of 120,000 as well as the intrinsically large evanescent field, allows for the implementation of a very compact spectrometer without the need for complex multi-pass gas cells. Water vapour concentrations as low as 4 parts per million at atmospheric conditions have been readily detected in proof-of-concept experiments.

## 1. Introduction

Terahertz (THz) radiation, located between microwave and infrared frequencies, bridges the gap between electronics and optics, and has attracted significant interest for broadband gas-phase spectroscopy. In particular, because of the abundance of strong fundamental absorption lines of a large variety of molecules in this frequency range [1,2,3,4,5,6,7,8,9]. However, current cutting edge THz gas-phase spectrometers still require cumbersome multi-pass gas cells to reach sufficient sensitivity for trace level gas detection [10,11,12,13]. In order to fully exploit the potential of THz gas-phase spectroscopy, novel sensors that are highly frequency selective and sensitive to changes in the surrounding medium are required. Here, we present the first demonstration of THz gas-phase spectroscopy by exploiting the unprecedented frequency selectivity and environmental sensitivity characteristic to sub-wavelengths thick ultrahigh quality factor (ultrahigh-Q) disc microresonators. The specifically designed ultrahigh-Q THz disc microresonators allow for the implementation of compact, highly sensitive THz gas-phase spectrometers.

**Figure 1:**
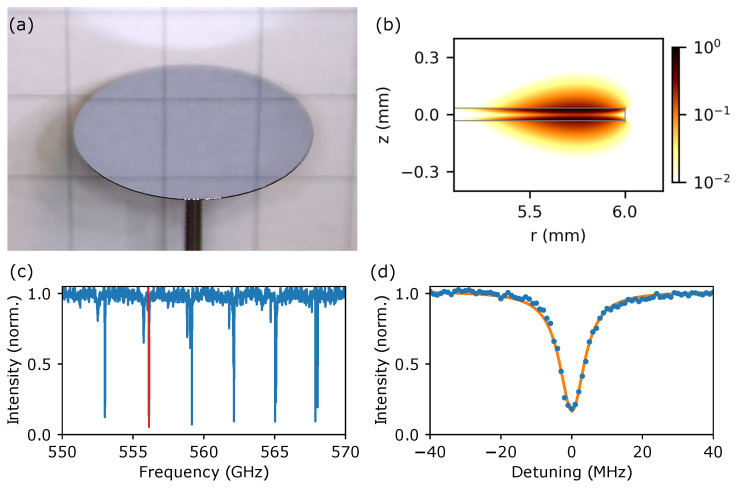
(**a**) Microscope image of a 12 mm diameter, 66 ± 1 μm thick HRFZ-Si THz disc microresonator. The resonator is mounted on a 1 mm diameter aluminium rod. (**b**) Corresponding simulated intensity distribution (normalised) on logarithmic scale showing the large extend of the evanescent field. The microresonator cross-section is indicated with grey lines. Please note that all simulations presented in this work are performed with COMSOL Multiphysics^®^ software [32], and fabrication imperfections are not considered in the simulations. (**c**) Measured intensity profile of the THz disc microresonator showing the fundamental mode. (**d**) Resonance at 0.5561 THz (highlighted in red in sub-figure (c)) close to critical coupling. The frequency step size is 1 MHz (blue dots). The fitted analytical model [33] is shown with the orange solid line.

Ultrahigh-Q microresonators are devices that can confine radiation with exquisitely low losses. Such low losses give rise to ultra-narrow resonance features that imply very fine frequency selectivity [14]. Moreover, even a slight change in the surrounding medium will perturb the resonances to a measurable extent, allowing for environmental sensitivity. To achieve sufficiently low losses (high Q-factors), the resonators must be formed from a single piece of material. While such monolithic resonators have been extensively studied at optical frequencies [15,16,17,18,19,20,21,22], they remain largely unexplored in the THz domain. It is only very recently that this concept has been translated into the THz domain [23,24,25,26,27,28,29,30,31], achieving unprecedented Q-factors more than two orders of magnitude higher compared to previous systems [29]. Unfortunately, while the demonstrated devices highlight the potential of monolithic THz resonators, their characteristics are still insufficient for demanding gas-phase spectroscopy applications. This is because in traditional resonator designs (e.g., a sphere, disc, or ring), the THz radiation resides predominantly inside the resonator, limiting the devices’ Q-factor and environmental sensitivity. We overcome this deficiency by exploiting the sub-wavelength confinement offered by a novel class of thin-disk microresonators, where the radiation is forced to reside outside of the resonator, as a large evanescent field (see Figure 1a,b). As a proof of concept, we demonstrate as an exemplar the detection of less than 4 parts-per-million by volume (ppmv) of water vapour at ambient conditions. Novel sensing schemes for minute water vapour concentrations are in immediate interest, for example, for lithium-ion battery manufacturing, semiconductor fabrication and pharmaceutical industries [11]. However, we envision that the presented compact THz gas-phase spectrometer with the ultrahigh-Q THz disc microresonator will enable a myriad of possibilities in, for example, medical or industrial applications.

## 2. Materials and Methods

Key to the THz disc microresonator’s unprecedented frequency selectivity and environmental sensitivity, apart from a low-loss substrate (here, high resistivity float-zone grown silicon), is the sub-wavelength thickness of the disc resonator. Thinning the disc to a fraction of the free-space wavelength shifts a large proportion of the THz radiation—that would otherwise be confined to the lossy resonator substrate—to the surrounding environment. This not only reduces the impact of the disc’s material absorption (increasing the Q-factor); but also intrinsically produces a large evanescent field, leading to a significant overlap with the resonator’s environment. Figure 1a,b show images of a fabricated sub-wavelength thick THz disc microresonator and the corresponding simulated intensity distribution. The fabricated microresonator has a 12 mm diameter and a thickness of 66 ± 1 μm, corresponding to $\lambda_{0}$/8 at 0.6 THz. The calculated evanescent field fraction *f* (ratio of the energy of the electromagnetic radiation outside of the resonator to the overall energy) of this disc is 52%. This compares to a calculated evanescent field fraction of about 0.3% for a 4 mm diameter spherical silicon THz microresonator, highlighting the enhancement in evanescent field of the sub-wavelength thick disc compared to a spherical resonator design.

As mentioned above, a low-loss resonator substrate is essential as even with a thickness of $\lambda_{0}$/8 about 50 % of the total energy remain in the disc’s substrate. Here, the disc microresonator is made of high resistivity float-zone grown silicon (HRFZ-Si) with a resisitivity of >10 kΩcm, as this is one of the lowest loss materials known in the THz domain [29]. Fabrication of the discs is achieved by thinning a standard 100 μm thick, 2 inch diameter wafer and subsequent laser micro-machining. Finally, the disc’s rim is polished using a fine diamond slurry to remove any imperfections from the laser micro-machining. The disc microresonator’s diameter and thickness are chosen to maximise the Q-factor and extent of the evanescent field at a specific design frequency—here about 0.6 THz according to the water vapour absorption line at 0.557 THz [34]. The detailed intricacies of the resonator design are outlined elsewhere [35].

The fundamental resonances of the disc microresonator are shown for the frequency range from 0.55 THz to 0.57 THz in Figure 1c as an example. The free spectral range (FSR) of the fundamental mode is about 3 GHz. Figure 1d shows the resonance at 0.556 THz, with an intrinsic (no coupling, in an ideal non-absorbing gas) Q-factor of about 120,000. Experimentally, the intrinsic Q-factor is obtained by fitting the measured Q vs ppmv dependence with an analytical model [33] and extrapolating to 0 ppmv (see detailed discussion below).

**Figure 2:**
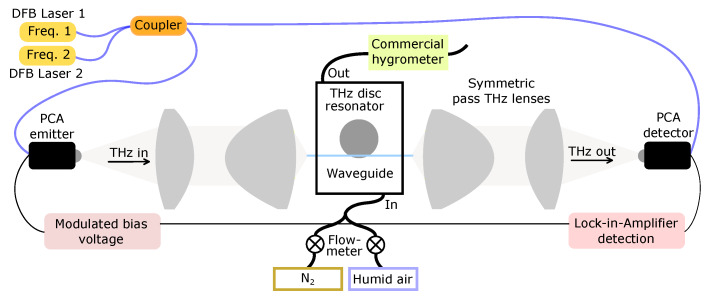
Schematic of the gas-phase THz spectrometer with a commercial CW-THz system and a sub-wavelenth thick THz disc microresonator. The THz microresonator is mounted on a 3D translation stage to control the position of the resonator relative to the air-flouropolymer-silica waveguide. Both the horizontal and vertical position of the waveguide relative to the resonator were monitored with digital microscopes. Because of the intriguing field distribution, best coupling is achieved by placing the waveguide above or below the edge of the disc. Strong coupling is typically achieved at a position of the waveguide of about 200 μm inside from the edge of the microresonator and a gap of about 100 μm–200 μm to the microresonator. The deployed symmetric-pass THz lenses are specifically designed to achieve high coupling efficiency to the sub-wavelength waveguide [39].

The resonances shown in Figure 1c are excited using evanescent coupling with a single-mode air-flouropolymer-silica step-index waveguide with a 200 μm silica core that is coated with a flouropolymer layer of 12.5 μm thickness. The waveguide provides single-mode operation in the frequency range from 0.4 THz to 0.65 THz, with an effective refractive index of 1.27 to 1.37 from 540 GHz to 600 GHz. The coupling strength of the resonance can be tuned by changing the distances between the waveguide and the microresonator. A schematic of the experimental setup is shown in Figure 2. The THz radiation is generated and detected using fiber-coupled photoconductive antennas (PCA) with a continuous-wave (CW) THz system from Toptica (TeraScan 1550 [36]). The smallest step-size of the CW-THz system is about 1 MHz, which is essential in sufficiently resolving the very narrow ultrahigh-Q THz resonances with a typical line width of less than 10 MHz. The data analysis is performed using a Hilbert transform, which is explained in great detail elsewhere [37,38]. The intensity profiles shown in Figure 1c,d are obtained by normalising a sample scan (waveguide transmission with the coupled resonator) with a reference scan (waveguide transmission without the resonator). This eliminates undesired effects, like standing waves in the setup and frequency-dependent performance of the THz antennas, thus solely provides the frequency response of the THz microresonator.

The operational principle for the gas-phase spectroscopy using the THz disc microresonator is to monitor a change in Q-factor under variation of the gas concentration in the resonator’s environment. This modality is known to be more sensitive than, for example, observing resonance frequency shifts [40], and takes advantage of the strong gas absorption lines in the THz domain. Of course, this requires the microresonator’s resonances to coincide with the desired gas absorption lines. The spectral position of the resonances is determined by the HRFZ-Si resonator’s geometry and can subsequently be tuned, for example, by using thermal mechanisms [41,42]. Here, the 12 mm diameter disc shown in Figure 1a has an ultrahigh-Q resonance at 0.5561 THz at room temperature which is sufficiently close to the water vapour absorption line at 0.5569 THz (with a line width of about 6 GHz), and therefore no tuning was performed.

The location of the gas-cell used in this proof-of-concept experiment is indicated in Figure 2. The gas-cell is milled from Perspex and features a rectangular design with two Teflon windows to ensure a high transmission of the THz signal. The windows are also used as mounts for the waveguide with the end-faces of the waveguide protruding to the outside of the gas cell. The gas-cell is designed to operate under a constant gas flow with the inlet and the outlet at opposite sides of the gas-cell. The humidity level is adjusted by controlling the flows of Nitrogen and humid air at the inlet of the gas cell, and monitored at the output using a high-end commercial hygrometer (Vaisala DMT152) which serves as a reference for the actual water vapour concentration. We note, that this proof-of-concept experimental setup is not suitable for field work, however, we envision, that in the future a more integrated approach will lead to a much lower susceptibility to vibrations. The experimental procedure is as follows: (1) Adjustment of the desired gas concentration and ensuring a constant gas flow/ppmv reading. (2) Performing a reference scan, that is, the waveguide transmission without coupling to the THz disc microresonator. (3) Adjust the distance between the waveguide and the resonator to achieve strong coupling and perform a sample scan. (4) Finally, the intensity and phase profiles obtained from the waveguide transmission are fitted simultaneously with the complex analytical model to obtain the resonator’s Q-factor at this specific gas concentration. Utilising intensity and phase information provides a higher confidence in the obtained Q-factor than solely relying on the intensity. Finally, steps (2) to (4) are repeated several times to further reduce the standard error in Q-factor.

## 3. Results and Discussion

**Figure 3:**
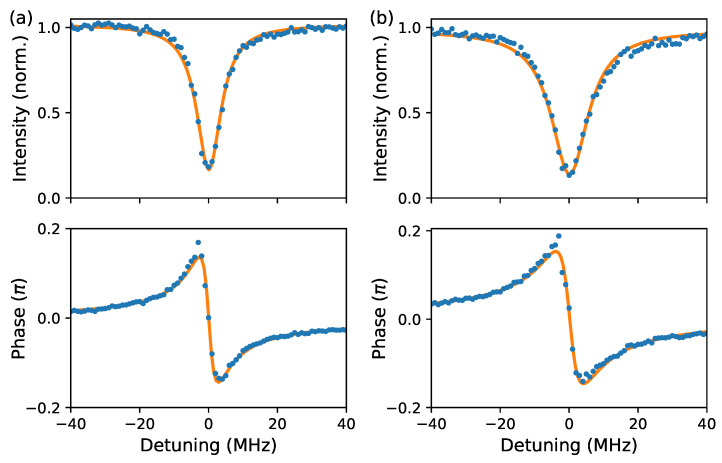
(**a**) Measured intensity and phase profiles (blue dots) of the resonance at 0.5561 THz at 7 ppmv with the corresponding fit (orange solid lines). (**b**) The same resonance at 120 ppmv water vapour concentration. Both measurements are recorded with similar coupling strength to ease comparison.

Figure 3 shows the measured intensity and phase profiles (blue dots) of the resonance at 0.5561 THz at (a) 7 ppmv and (b) 120 ppmv water vapour concentrations. The fitted analytical model (orange solid lines) is in very good agreement with the measurements. Comparison of Figure 1a,b shows the broadening (lower Q-factor) of the investigated resonances due to the increased water vapour absorption, and nicely demonstrates the principle of the sensing modality.

**Figure 4:**
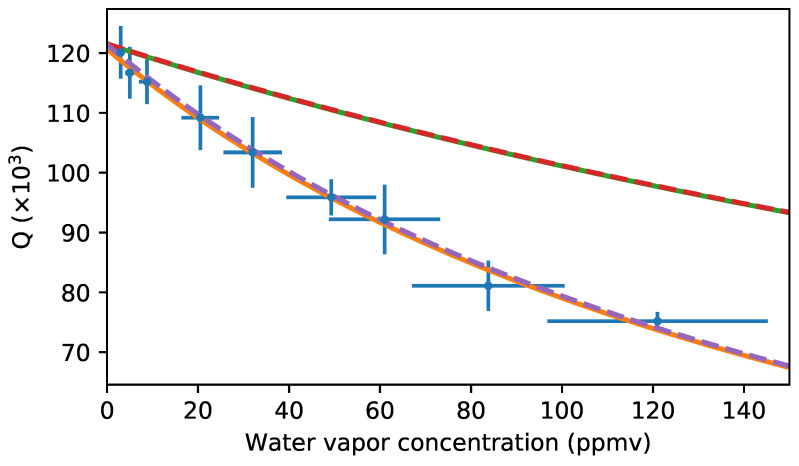
Measured Q-factors (blue dots with error-bars) as a function of water vapour concentration, with the corresponding fit (Equation (1), orange solid line). The calculated and simulated Q (ppmv) curves using the HITRAN database are shown with a green solid and red dashed lines, respectively. The simulated curve assuming a continuously growing water layer film on the disc is shown with the purple dashed line. The water layer is modelled as a Transition Boundary Condition with a uniform coverage of the disc, and an effective layer thickness. The dielectric function assumed for the liquid water layer is ϵ=4.8+i3.2 [45].

The measured Q-factors (blue dots) for a range of water vapour concentrations from 4 ppmv to 120 ppmv (0.01–0.49% relative humidity) are summarised in Figure 4. The horizontal error-bars show the uncertainty in the ppmv reading as calculated from the specified ±2 °C uncertainty in the frost point temperature displayed by the commercial hygrometer. An uncertainty of ±2 °C in frost point temperature converts to about 20% to 30% uncertainty in the ppmv reading in the range from 4 to 120 ppmv. This large uncertainty is typical for a high-end commercial hygrometer in this concentration range. While this somewhat limits the ability to calibrate the presented THz spectrometer with sub-wavelength thick THz disc microresonator, it also reveals the persisting challenges in the field of low water vapour concentration detection, and the need for better technologies. The vertical error-bars result from the uncertainty in the Q-factor extracted from the analytical model fitted to the measurements. A main limiting factor in the uncertainty of the fitted Q-factor is the frequency resolution and stability of the deployed CW-THz System. A higher frequency resolution/stability, as for example with a comb-locked frequency-domain terahertz spectrometer [43], would greatly reduce the uncertainty, and ultimately improve the capability to distinguish small changes in the Q-factor.

The change in Q-factor as a function of the water vapour concentration can be modelled by the following analytic equation:

(1) $$Q=\frac{C}{\alpha_{i}+f\alpha_{\mathrm{gas}}\left(\nu_{0},ppmv\right)}.$$

With $\alpha_{i}$ the effective resonator loss (includes material loss, radiation loss, scattering loss etc.) surrounded by a lossless gas, and $\alpha_{\mathrm{gas}}\left(\nu_{0},ppmv\right)$ the gas absorption at the resonator resonance frequency $\nu_{0}$ as a function of water vapour concentration. *C* is a constant defined as *C* = 2π$n_{s}$$\nu_{0}$/c, with $n_{s}$ the refractive index of the resonator substrate ($n_{\mathrm{HRFZ}-\mathrm{Si}}$ = 3.416 at 0.6 THz), and c the speed of light [33]. This functional dependence has been used in Figure 4 to fit the solid orange curve to our data points, and the trend of the data agrees very well with the analytic expression. For a more quantitative comparison $\alpha_{gas}\left(\nu ,ppmv\right)$ can be expressed using the HITRAN-database notation as follows [34]:

(2) $$\alpha_{gas}\left(\nu ,ppmv\right)=NSg\left(\nu \right)p.$$

With *N* the total number of molecules of absorbing gas, S is the molecular line intensity, g(ν) the absorption line profile and *p* the gas partial pressure, which can be related to the ppmv via $ppmv=p/p_{\mathrm{atm}}10^{6}$, with $p_{\mathrm{atm}}$ the total pressure in the gas cell (here typically 1 atmosphere). For the absorption line profile g(ν) we are using the van-Vleck Weisskopf profile which is most suitable for the microwave/terahertz domain [44].

Interestingly, while the experimental data follows the trend predicted by Equation (1), the calculated Q vs. ppmv dependence (combining Equations (1) and (2)) using the values from the HITRAN database (green solid curve) produces a curve that indicates lower absorption than the experimental results. However, the calculated and simulated (red dashed line) Q vs ppmv dependence are in excellent agreement, confirming the analytical approach described above. A possible explanation for this discrepancy could be the presence of a thin layer of liquid water adsorbed to the surface of the microresonator. The adsorbance of water is a ubiquitous process [46], and has been previously reported to reduce the ultrahigh Q-factor of microresonators in the optical domain [47,48]. Here, for example, at 80 ppmv we get quantitative agreement with the experiments if we assume a uniform coverage of the disc with a water layer of about 3 *Å* thickness, corresponding to about 1 mono-layer of water [46]. While this is a relatively thick coverage of the disc, at the given water vapour concentration, in comparison to reports in the literature, an increase in layer thickness from 1.5 *Å* at 40 ppmv to 3 *Å* at 80 ppmv would perfectly explain our results (see purple dashed curve in Figure 4). While there is undoubtedly water adsorbed to the THz disc microresonator, unfortunately we are not able to provide any further evidence about the thickness of the liquid water layer, as we have no other means to determine the thickness.

Nevertheless, the experimental results clearly show that the THz disc microresonator with sub-wavelength thickness can detect water vapour concentrations as low as 3 ppmv by interrogation of the absorption line at 0.5569 THz. Noteworthy, the sensitivity of the THz disc microresonator solely originates from the large evanescent field, and has not been enhanced using humidity absorbing materials like silica gel or surface activation [49,50]. Consequently, the reduction in Q-factor is immediate and shows no hysteresis as is common for many commercial hygrometers.

In general, due to the proportionality of the Q-factor to the inverse of the gas absorption (see Equation (1)), the microresonator shows the highest sensitivity at gas vapour concentrations leading to Q-factors close to the intrinsic Q-factor. Furthermore, the higher the intrinsic Q-factor, the steeper the drop-off in Q-factor with increasing gas concentration. Considering the experimental results presented in Figure 4, the best operation range for this resonator is estimated to be in the range from 0 ppmv to 1000 ppmv (0–4% relative humidity) water vapour concentration. In order to cover a wider range of water vapour concentrations, a combination of THz disc microresonators with various intrinsic Q-factors could be envisioned (with a lower/higher intrinsic Q-factor for higher/lower water vapour concentrations). Finally, the regularly spaced resonances over a broad frequency range (up to 200 GHz, see for example, Figure 1c, render the sub-wavelength thick THz disc microresonators ideal for the detection of gas mixtures (provided that the resonances coincide with specific gas absorption lines).

## 4. Conclusions

The presented results clearly demonstrate the sensing capabilities of the gas-phase THz spectrometer utilising the ultrahigh-Q THz disc microresonator. Minute water vapour concentrations in the ppm concentration level are detected without the need for a complex and bulky multi-pass absorption cell. In particular, the experimental data agrees very well with a calibration curve provided by a high-end commercial hygrometer. Additionally, perfect quantitative agreement is achieved by considering a few angstrom thick layer of liquid water adsorbed to the ultrahigh-Q THz disc microresonator. Ultimately, even higher sensitivities are within reach by further optimising the resonator design and utilising THz spectrometers with higher frequency resolutions, like a comb-locked frequency-domain spectrometer. The presented results are alluding to the possibility of compact and highly sensitive THz gas-phase spectrometer contributing to a wide range of applications.
